# Polarization-Independent and Electrically Tunable Polymerized Liquid Crystal Optical Elements

**DOI:** 10.1021/acsphotonics.5c01416

**Published:** 2025-12-09

**Authors:** Zhiyu Xu, Camron Nourshargh, Waqas Kamal, Alec Xu, Steve J. Elston, Martin J. Booth, Stephen M. Morris

**Affiliations:** Department of Engineering Science, 6396 University of Oxford, Oxford OX1 3PJ, U.K.

**Keywords:** direct laser writing, two-photon polymerization, nematic liquid crystals, polarization insensitive

## Abstract

In this paper, we demonstrate laser-written polymerized liquid crystal (LC) diffractive-optical elements that combine polarization-independent operation with real-time electro-optic tuning. The study explores the design, simulation, fabrication, and characterization of several different polarization-independent optical elements, including diffraction gratings, Fresnel zone plates, and holograms. Leveraging two-photon polymerization direct laser writing, these polarization-independent diffractive optic elements were realized through stacked configurations, ensuring functionality even for unpolarized light conditions. The tunable and switchable nature of these LC optical elements supports dynamic imaging capabilities, including vari-focal functionality, allowing multiple focal planes for enhanced depth perception. The polarization-independent and vari-focal properties make these optical components highly desirable for next-generation applications in immersive display systems, addressing challenges such as compact form factor and visual fatigue. Additionally, the thin, lightweight design and high optical efficiency of these elements make them highly desirable for integration into adaptive optics, holographic displays, and other advanced optical technologies.

## Graphical abstract

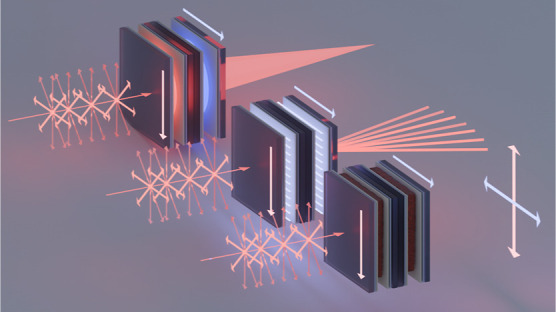

## 1 Introduction

Liquid crystals (LCs), which combine fluid-like properties with a degree of long-range molecular order, are key functional materials for tunable photonic devices due to their remarkable electro-optic characteristics and anisotropic optical response.^1^ By carefully controlling the orientation of the LC director (the average pointing direction of the molecules) via external fields, alignment layers, and/or patterned surfaces, it is possible to modulate the polarization, phase, and direction of incident light, thus enabling the realization of dynamic optical elements such as phase retarders, display devices, and spatial light modulators (SLMs).^2–4^

Optical wavefront control is essential in various scientific and engineering fields, enabling the precise manipulation of light for imaging, fabrication, and display technologies. Reconfigurable phase modulators such as SLMs and deformable mirrors (DMs) play a central role in these applications, offering programmable adjustment of the optical phase profiles. In microscopy adaptive optics, SLMs have been used in techniques such as confocal and multiphoton imaging to correct for image distortions caused by system- or specimen-induced aberrations, thereby enhancing image quality.^5^ In micro-fabrication, SLM-generated holograms are used to simultaneously generate multiple focal spots to facilitate parallel processing, enabling faster throughput. These capabilities have proven crucial in fields ranging from adaptive optics to holographic displays, underscoring the flexibility and importance of dynamic phase control.^6–8^

Conventional LC SLMs are pixelated devices, which enable independent phase control at each individual pixel and are essential for holographic and beam shaping applications as mentioned earlier.^9^ However, due to the pixelated design, fill factor loss needs to be considered. This dead zone will not manipulate incident light, causing a lower photon efficiency. Also, the discrete phase steps between adjacent pixels act as a diffraction grating, giving rise to unwanted higher diffraction orders and speckle artifacts that degrade image quality and contrast. In addition, driving a large number of pixels requires complex electronics, increasing the system size and cost. While these limitations are not the primary focus of this work, our laser written fabricated device that is presented in this paper inherently provides a continuous, nonpixelated phase profile, offering a simpler optical architecture while addressing the more critical challenge of polarization independence.

The key limitation of LC SLMs, unlike DMs, lies in their polarization dependence. Due to the parallel alignment conditions used in conventional nematic LC phase modulators, they only modulate the phase of light for linear polarization, parallel to the LC alignment axis. Typically, this issue is addressed in two ways: (1) by employing a deformable mirror (DM) – which adds cost and usually offers a smaller phase range, fewer pixels, and lower spatial resolution than SLMs – or (2) by using multiple SLMs in the beam path to modulate each polarization state separately. Without this modification, half of the light (the unmodulated polarization component) was filtered out before detection. In low light intensity scenarios, such as fluorescence imaging, this presents a serious challenge, and this inefficiency is also particularly detrimental in applications demanding high throughput and compact form factors, such as augmented reality (AR), virtual reality (VR) headsets, as well as high-resolution microscopy, where maximum photon efficiency is critical. Furthermore, both LC SLMs and DMs tend to be expensive and involve complex drive electronics, making them less suitable for portable devices that require simpler, cheaper, and smaller form factors.

Previous work has reported several different approaches to forming switchable, polarization-insensitive LC phase modulators. For example, early demonstrations of both single layer and double layer polarization-independent LC phase modulators have been reported,^10–13^ providing the foundation for subsequent development in this field. Using only a single-layer, one approach is to employ optically isotropic or blue phase LCs, rather than conventional nematic LCs, as these would enable polarization-insensitive modulation in a single layer.^14^ These are, however, notoriously difficult to form and stabilize at room temperature.^15^ More recently, Tang et al. utilized the twisted nematic configuration to enable switching of both polarization states from a single LC layer. While overcoming the issue of polarization sensitivity, both of these approaches still require a pixelated device to drive them, increasing the bulk and complexity of associated electronics, which will limit the form factor of the final device, preventing easy integration into compact systems.^16^

A more advanced strategy demonstrated by He et al. employs two-photon polymerization (TPP) to fabricate a double-layer LC architecture with orthogonal alignments separated by an ultrathin polymeric partition,^17^ thereby enabling polarization-independent phase modulation. While this approach eliminates the need for pixelated addressing and achieves a full 2π phase shift at low voltage, the fabrication process is relatively complex, requiring submicron resolution 3D nanostructuring and precise control of multilayer alignment, which may present challenges for scalability and mass production.

In this work, we present an alternative approach for realizing switchable, polarization-insensitive phase modulators that operate using simple drive electronics and have a compact transmissive form factor. To overcome the issue of complex drive electronics, we also use two-photon polymerization direct laser writing (TPP-DLW) to produce fixed polymer structures in the LC (Figure 1) within the bulk rather than as a separate layer. TPP-DLW is a microfabrication technique that allows the fabrication of 3D structures in polymer resins at submicron length scales. As in our previous work, TPP-DLW was used to fabricate structures into the LC layer, fixing the LC director orientation in the polymerized regions, while the nonpolymerized regions remained electrically tunable.^18,19^

**Figure 1:**
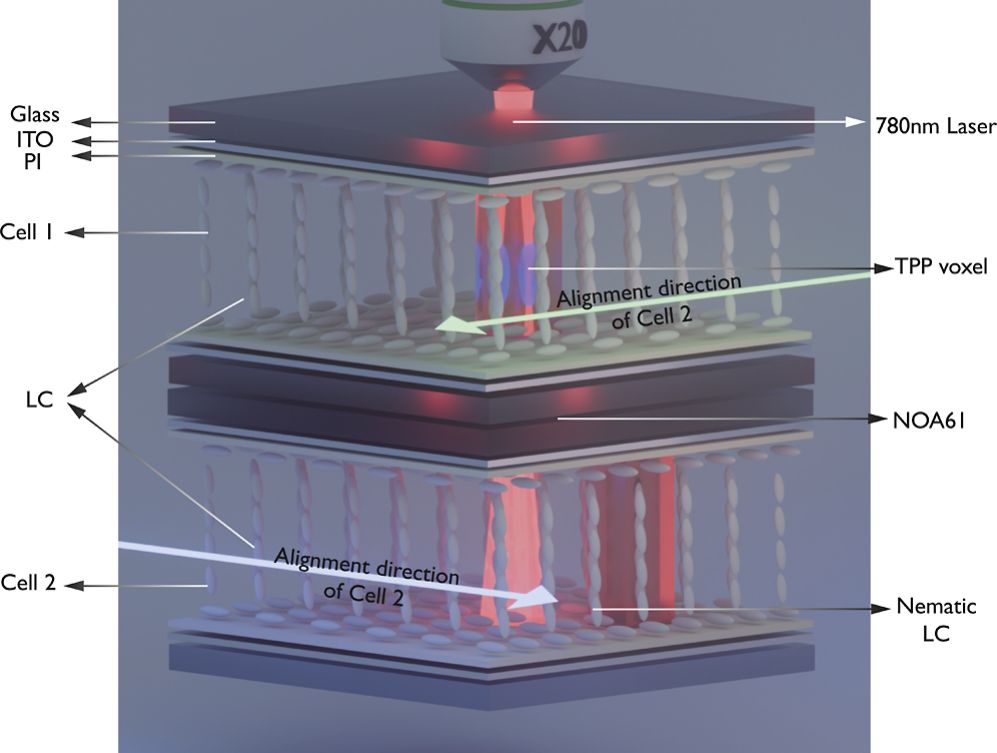
A schematic illustration showing the concept of stacked laser-written liquid crystal (LC) devices for polarization-insensitive and polarization-sensitive functionality. Two glass cells with separate LC layers are stacked one on top of the other. TPP-DLW is then used to fabricate high fidelity polymer structures in polymerizable LC layers in cell 2 and then cell 1. The solid red regions indicate the polymer network formed that locks in the director.

While the devices produced using TPP-DLW are still polarization-sensitive, this limitation can be overcome by stacking a pair of LC glass cells with their rubbing directions arranged orthogonally, as shown in Figure 1. In this way, each cell interacts with orthogonal polarization states, resulting in polarization-insensitive operation for the complete device if both structures are fabricated and driven in the same way. Alternatively, a polarization-selective operation can be achieved if the polymer structures or voltage driving conditions differ. Here, we demonstrate the versatility and robustness of the stacked architecture by fabricating three different representative optical elements: diffraction gratings, binary Fresnel zone plates (FZP), and holographic phase patterns. These devices highlight the capacity to produce either polarization-independent focusing and diffraction (when both cells share the same structure) or polarization-selective outputs (when each cell is encoded with a distinct phase pattern). Our results confirm that the stacked arrangement mitigates the polarization sensitivity inherent in single-layer LC systems without resorting to the use of external polarizers or complex multielement designs. This paper presents the device architecture, the TPP-DLW fabrication process, and the experimental evaluation of three different LC devices (gratings, binary FZPs, and on-demand holographic phase patterns), realized in orthogonally aligned stacked glass cells to achieve either polarization-independent or polarization-selective responses.

## 2 Results and Discussion

Each type of device (e.g., diffraction gratings, FZP, and holograms) was fabricated in a stacked pair of LC glass cells (Figure 1). The exact fabrication conditions and the configurations of the glass cells are explained in more detail in the Experimental Section. In the following three sections, results are presented for each of the three different types of device, starting with the diffraction gratings. For clarification, the polymer network structures were written when the LC devices were subjected to an applied voltage of 20 Vpp, resulting in a homeotropic alignment of the LC director inside the polymer network. From hereon, the term *switched ON* refers to the application of a moderate applied voltage (3.5 Vpp) to the LC device where the nonpolymerized LC region is free to switch, resulting in a phase difference across the device. The term *switched OFF* refers to the application of a higher voltage (20 Vpp) to ensure a uniform homeotropic alignment of the LC, resulting in no phase difference across the device. We further demonstrate that our stacked polymerized LC layers can also achieve a continuous phase modulation range approaching a full 2π under low-voltage operation (see Supporting Information Figure S1).

### 2.1 Diffraction Gratings

Diffraction gratings are versatile optical components that underpin numerous technologies for precise spectral discrimination, wavefront control, and beam steering.^20,21^ Their capacity to operate over a wide spectral range, extending from the ultraviolet to the infrared, further broadens their utility in scientific research, industrial instrumentation, advanced communications, and next-generation display technologies.^22–24^ The well-known Bragg equation, *m*λ = *d* sin θ_m_, describes the constructive interference conditions for a diffraction grating, where *m* is the diffraction order, *d* is the grating period, and θ_m_ is the diffraction angle. Here, we are specifically interested in the design and fabrication of a binary diffraction grating designed to function at a wavelength of 632 nm (He–Ne laser) that is insensitive to the incident polarization state.

Figure 2a presents a schematic illustration of the device architecture and the diffraction gratings that were laser-written into two stacked LC devices at a voltage of 20 Vpp (see Methods). The same diffraction grating patterns were fabricated into both cell 1 and cell 2 with the exact same parameters and relative locations within each LC layer. These gratings consist of periodic polymer walls that have been written in to lock the director in a homeotropic alignment. Figure 2a shows an illustration of the configuration of the diffraction gratings in the two sequential LC layers. In Figure 2b, a polarization optical microscope (POM) image shows the polymer walls fabricated by TPP-DLW at a voltage of 20 Vpp to form the diffraction grating. The sample was oriented at an angle that was slightly less than 45° relative to the orientations of the crossed polarizers for better visualization. The darker lines indicate that the director was oriented homeotropically and locked by a polymer network. The design of the grating, shown in Figure 2c, shows the spatial distribution of the light and dark regions, which agrees well with the fabricated grating as observed in the POM image.

**Figure 2:**
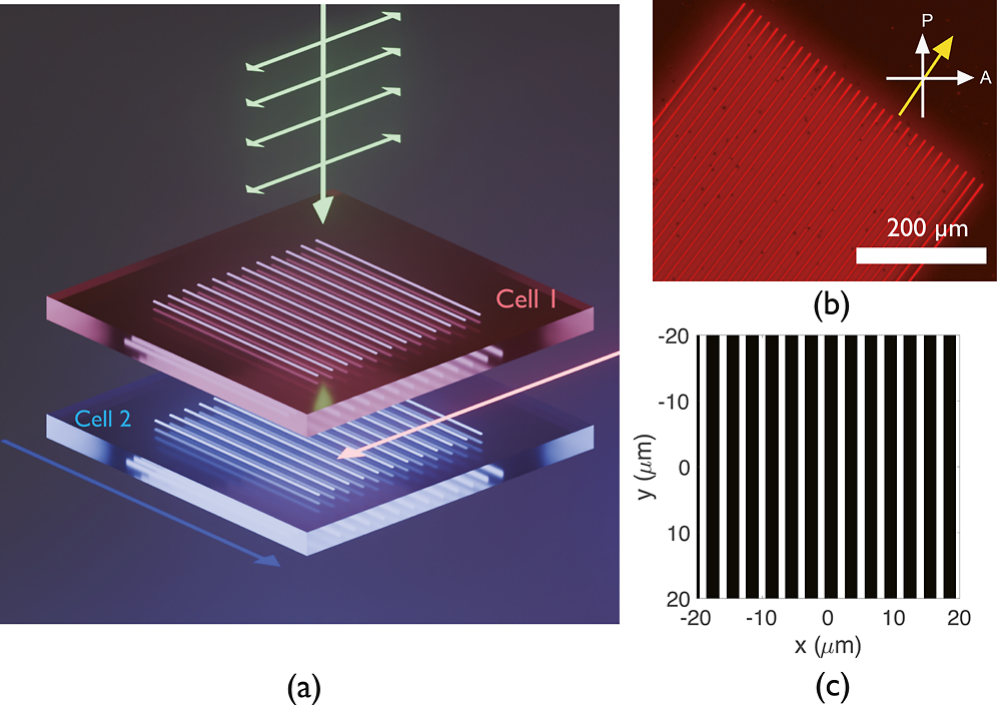
(a) Illustration of the device architecture indicating the orientation of the diffraction gratings and rubbing directions for the two stacked cells. The pink and blue arrows indicate the rubbing directions of the alignment layers for cell 1 and cell 2, respectively. (b) A POM image of the laser-written polarization-insensitive diffraction grating device. The single-headed white arrows indicate the orientations of the polarizer (P) and analyzer (A), while the single-headed yellow arrow represents the rubbing direction of the alignment layer. The scale bar is 200 μm, and the image was taken using a red long-pass band filter after the microscope light source to avoid postpolymerization. (c) The design of the diffraction gratings, where the white lines represent the laser-written areas, where the LC director is locked in place during the TPP process.

Figure 3 presents results for the far-field diffraction patterns when the gratings were switched either ON or OFF for three different incident polarization conditions. For these measurements, the stacked LC device was mounted in the experiment system, as described in the Methods. To begin, Figure 3a shows the case when both gratings were *switched OFF* with a voltage of 20 Vpp applied to both LC layers so that the director in the regions between the locked-in polymer walls was free to reorient to align with the electric field direction, thus removing the periodic modulation in the refractive index. As can be seen, there was only one bright spot at the center of the image in both the simulation and experiments, indicating that no diffraction occurred. With both devices *switched ON* using a lower voltage of 3.5 V, as is shown in Figure 3b, and the incident laser polarization angle set to θ = 0° (i.e., parallel to the rubbing direction of cell 1), a horizontal array of diffraction spots was observed on the camera. This is because the incident light experienced the diffraction grating present in cell 1 but not in cell 2. For cell 2, the incident polarization was aligned orthogonal to the rubbing direction, and so the light did not experience a periodic modulation of the refractive index.

**Figure 3:**
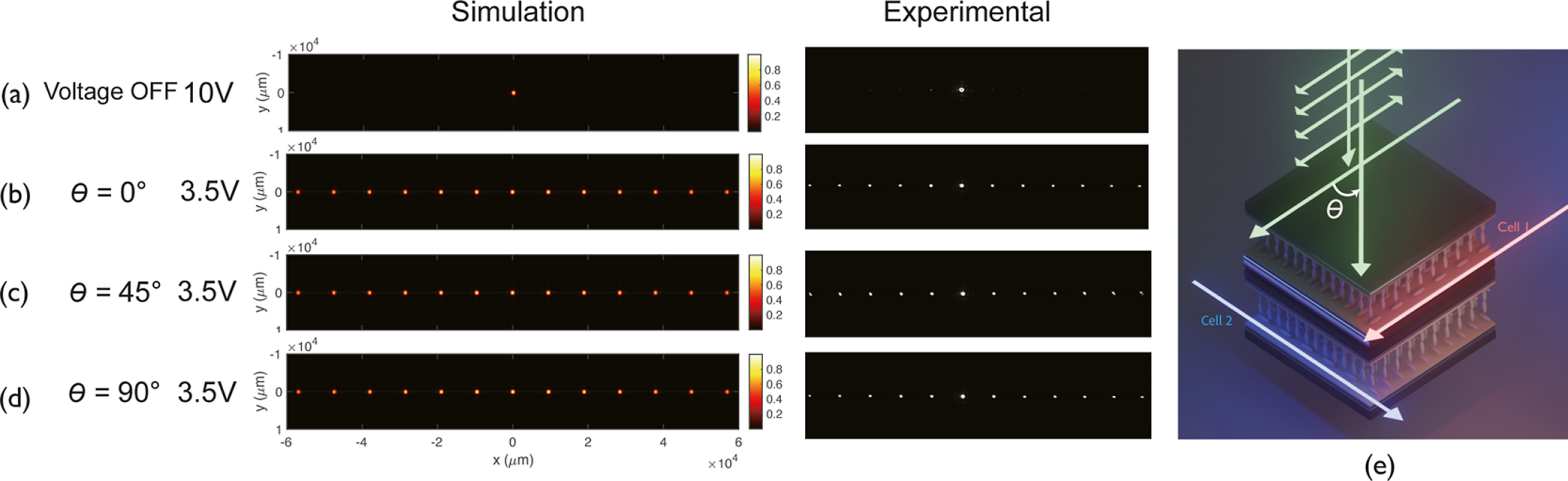
Far-field diffraction patterns recorded when two laser-written LC diffraction gratings were stacked together with the rubbing directions aligned orthogonally. Images are shown (a) the gratings were *switched OFF* using a large applied voltage (20 V) and at an applied voltage of 3.5 V for three different orientations of the incident laser polarization with respect to the rubbing direction of cell 1: (b) θ = 0°, (c) θ = 45°, and (d) θ = 90°. (e) A schematic illustration of the stacked LC device where the green arrows are the light propagation direction, the pink arrow is the rubbing direction of cell 1, and the blue arrow is the rubbing direction of cell 2. The double-headed arrows indicate the polarization direction.

Subsequently, when the polarization state of the incident laser was set to θ = 45°, effectively splitting the polarization of the incident light into two orthogonal vectors, the diffraction pattern appears to be unchanged, as shown in Figure 3c. For a single LC grating, a rotation of the incident polarization would cause the diffraction pattern to change. However, in this case, the diffraction pattern is maintained by a combination of both cell 1 and cell 2. Then, for a linear polarization oriented at θ = 90° Figure 3d, only cell 2 modulated the phase of the light, because the incident polarization was now aligned with the rubbing direction of cell 2. The grating in cell 1, in this case, was effectively inactive. Here, a clear diffraction pattern appeared, which would not be the case for a single LC layer and a single diffraction grating.

While the simulated diffraction pattern assumes an ideal phase distribution, the experimentally measured intensities exhibit small variations across the diffraction orders. This is expected, as the phase modulation produced by the stacked LC–polymer structures is not perfectly identical to the designed profile. Several practical factors may contribute to this deviation, including slight nonuniformities in the polymerized microstructures, small misalignment between the stacked LC layers, and a phase modulation depth that does not fully reach the theoretical value. As a consequence, the optical power is not distributed equally among the diffraction orders, as is the case for the simulated diffraction patterns.

Efficiency enhancement is a critical performance metric for stacked LC optical elements (LCOEs), as it determines how effectively incident light can be redistributed into target diffraction orders. To quantitatively assess this, we measured the *absolute* diffracted optical power and the *relative diffraction efficiencies* (ratio of diffracted order power to total transmitted power) for the zeroth to ±5 diffraction orders under different driving configurations (*switched ON/OFF*) of the stacked LC cells (see Supporting Information Figure S2). At an incident polarization angle of θ = 45°, the absolute optical power in the +first diffraction order increased from 0.62 μW (only cell 1 *switched ON*) to 1.12 μW when both cell 1 and cell 2 were *switched ON*, corresponding to a relative efficiency improvement of nearly 80%. Moreover, the total diffracted power was measured to be 6.45 μW (total diffraction efficiency of 37.76%). Importantly, this improvement is achieved without requiring input polarization control, providing direct experimental evidence that stacked LCOE architectures are capable of modulating *unpolarized* light, thereby overcoming a key limitation of single-cell LC diffractive devices.

Polarization-dependent measurements were also performed to evaluate the stability of the diffracted intensities in the stacked device configuration (Supporting Information Figure S3). The intensities of the ±1 and ±2 diffraction orders were recorded as the incident linear polarization was rotated from 0° to 180°. All orders exhibited only small fluctuations, with a maximum variation of approximately 20%, indicating that the overall phase modulation of the device is largely insensitive to polarization. This weak dependence might exist because both LC cells contribute to the total phase profile; however, slight differences in their alignment accuracy or residual birefringence can lead to a minor imbalance between the two cells. As a result, the diffraction orders do not remain perfectly constant with polarization but the deviation is minimal. These results confirm that the stacked structure provides effective polarization-tolerant performance suitable for applications where the incident polarization state is not strictly controlled.

By examining these specific polarization states whereby the individual gratings (cells) only manipulated the light at either θ = 0° or θ = 90°, and both cells combined simultaneously to form a diffraction pattern for an incident polarization of θ = 45°, as well as stepped polarization dependence measurements, we confirmed that the stacked LC diffraction grating achieved polarization-independent operation. The consistent diffraction patterns observed across all tested polarization states provide strong evidence that our stacked device maintained full functionality irrespective of the input polarization, successfully demonstrating the polarization independence of the system using diffraction gratings as an illustrative example.

### 2.2 Binary FZPs

In this section, we demonstrate how the concept of stacked laser-written LC elements can be combined to form tunable FZPs, which are binary phase devices designed to focus light. FZPs are formed of a series of concentric rings of increasing radii, with a π phase difference between each subsequent ring. The device can be designed to operate at a given focal length using the equation

1 $$r_{n}=\sqrt{n_{zones}\lambda f+\frac{1}{4}n^{2}\lambda^{2}}$$

where *r*_*n*_ is the radius of the *n*th ring, *n*_zones_ is the number of zones, λ is the wavelength of the incident light, and *f* is the designed focal length. A stack of laser-written FZPs is presented in Figure 4, where POM images of the FZPs in both cell 1 and cell 2 can be seen in Figure 4a, and the corresponding design is shown in Figure 4b. A 3D illustration of the stack is provided in Figure 4c.

**Figure 4:**
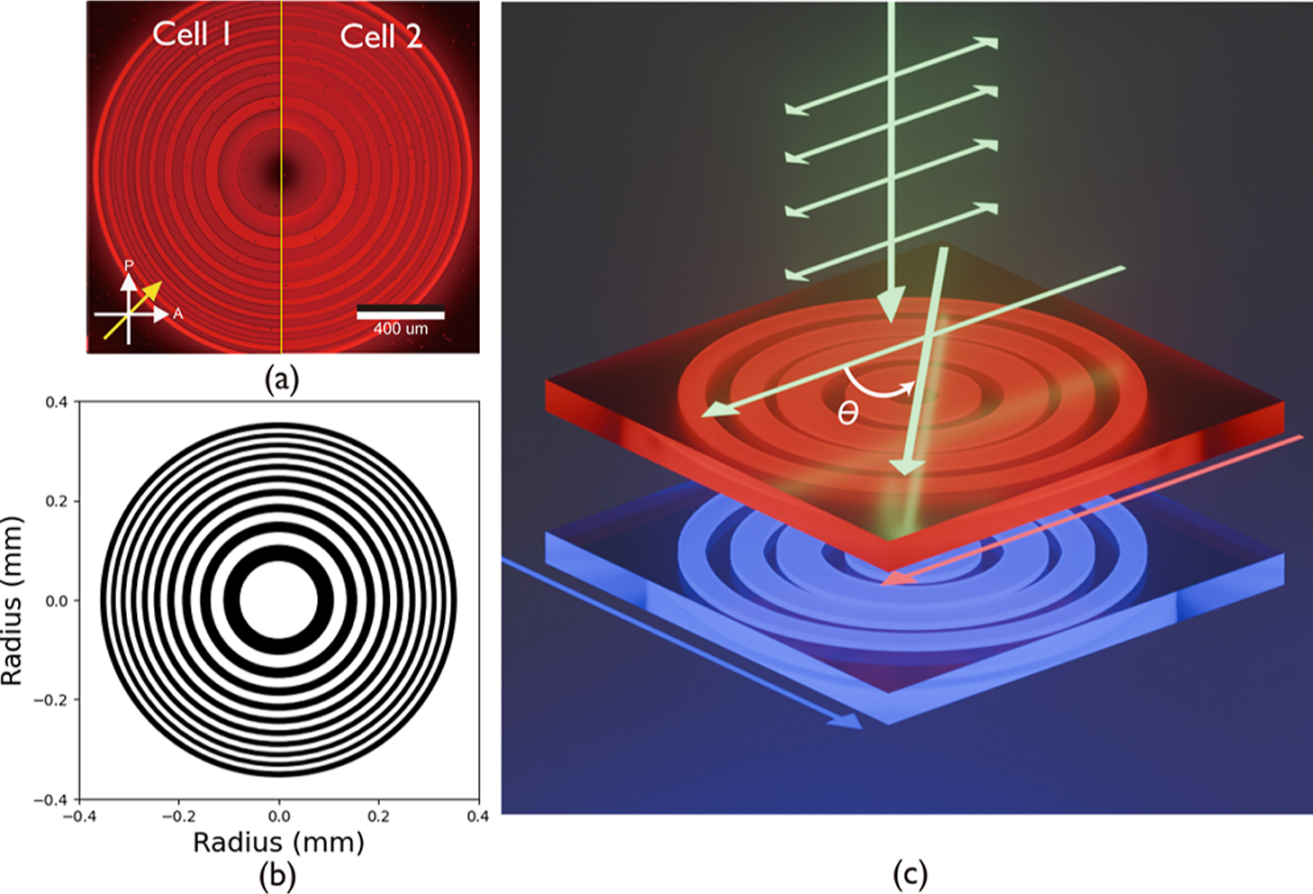
Polarization-independent FZPs. (a) Representative POM images of the fabricated binary FZPs in cell 1 and cell 2. (b) The design of the FZPs. (c) A 3D schematic illustration of the stacked FZPs. The pink arrow indicates the alignment direction of cell 1, and the blue arrow indicates that of cell 2.

Following fabrication, the stacked FZPs were then mounted on the sample holder in the characterization system (see the Methods). Figure 5 shows the far-field images of the focal spot for each cell and their combination, when the devices were either *switched OFF* (Figure 5a) or *switched ON* for a range of incident polarization states as shown in Figure 5b–d. It can be seen that the stacked devices could be individually controlled. For example, when only cell 1 was *switched ON* and cell 2 was *switched OFF*, for incident light with polarization θ = 0°, a clear bright spot could be seen at the center of the image, indicating that the focusing effect originated from cell 1.

**Figure 5:**
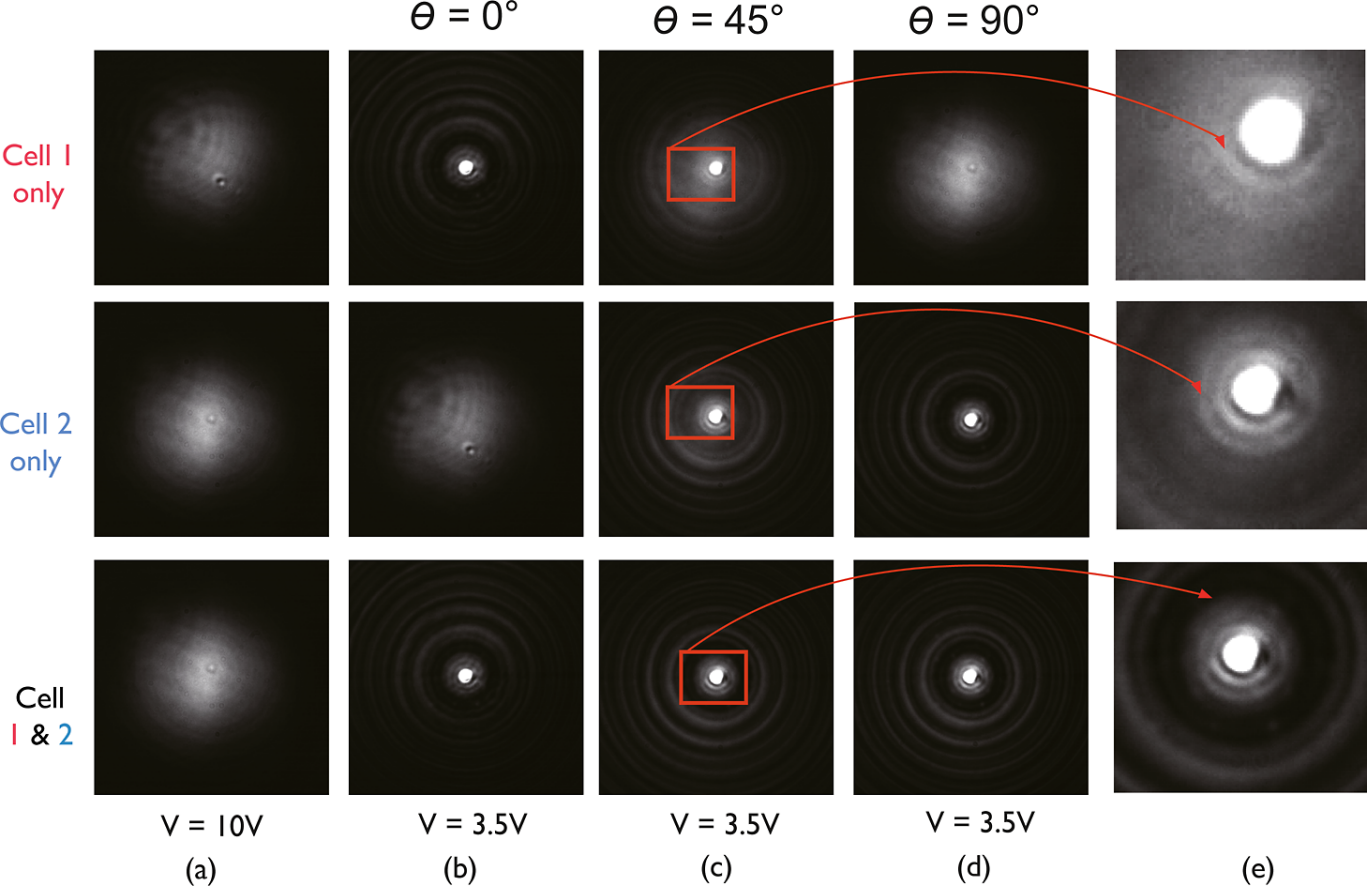
Images of the focusing of the stacked laser-written FZPs for different applied voltages and incident polarization states. Images were taken for the case when cell 1, cell 2, or both cells were (a) *switched OFF*. Images taken for the case when cell 1, cell 2, or both cells were *switched ON* with an applied voltage of 3.5 V for an incident polarization state that was aligned at the following angle relative to the rubbing direction of cell 1: (b) θ = 0°, (c) θ = 45°, and (d) θ = 90°. (e) Zoomed-in images of (c) for an incident polarization state aligned at θ = 45° relative to the rubbing direction of cell 1.

When the polarization of the laser was set to θ = 45°, its electric field could be considered as a vector sum of two orthogonal linear light polarization. In this case, both cell 1 and cell 2 could be used to manipulate the light, as cell 1 and cell 2 had optical axes parallel to the decomposed linear polarized vectors, respectively, as shown in Figure 5b. When only cell 1 was *switched ON* at θ = 45°, only the polarization state that was aligned along the rubbing direction of cell 1 could be used to focus light, and 50% of the incident laser remained unmodulated, and vice versa when only cell 2 was *switched ON*. This was confirmed by comparing the images for cell 1 and cell 2 *switched ON* at the same time. As shown in Figure 5e, when cells 1 and 2 were individually *switched ON*, a blurry pattern could be seen near the focal spot. However, when both cells were *switched ON*, a clearer bright spot without any blurring could be seen, confirming that stacking the LC FZPs/Cells with orthogonal rubbing directions could be used regardless of the incident polarization states.

When the polarization state of the incident light was set to 90°, no focusing could be seen when only cell 1 was *switched ON*, as all of the light was polarized along the rubbing direction of cell 2, which was *switched OFF*. This behavior was reversed when only cell 2 was *switched to ON*. In this case, we could confirm the polarization sensitivity of individual cells. Finally, when both cells were effectively *switched ON* by applying low voltages (1 V), they showed a clear bright spot for all three incident polarization states.

These switchable FZP lenses can be used in conjunction with a fixed glass lens to yield varifocal behavior. By placing a fixed lens as close to the input of the FZP as possible, as shown in Figure 6, the optical elements could be considered as a single component, and the subsequent focal length (*f*) can be calculated using

2 $$\frac{1}{f}=\frac{1}{f_{1}}+\frac{1}{f_{2}}-\frac{d}{f_{1}f_{2}}$$

where the fixed and LC FZP lenses have focal lengths of *f*_1_ and *f*_2_, respectively, and are separated by a distance *d*.

**Figure 6:**
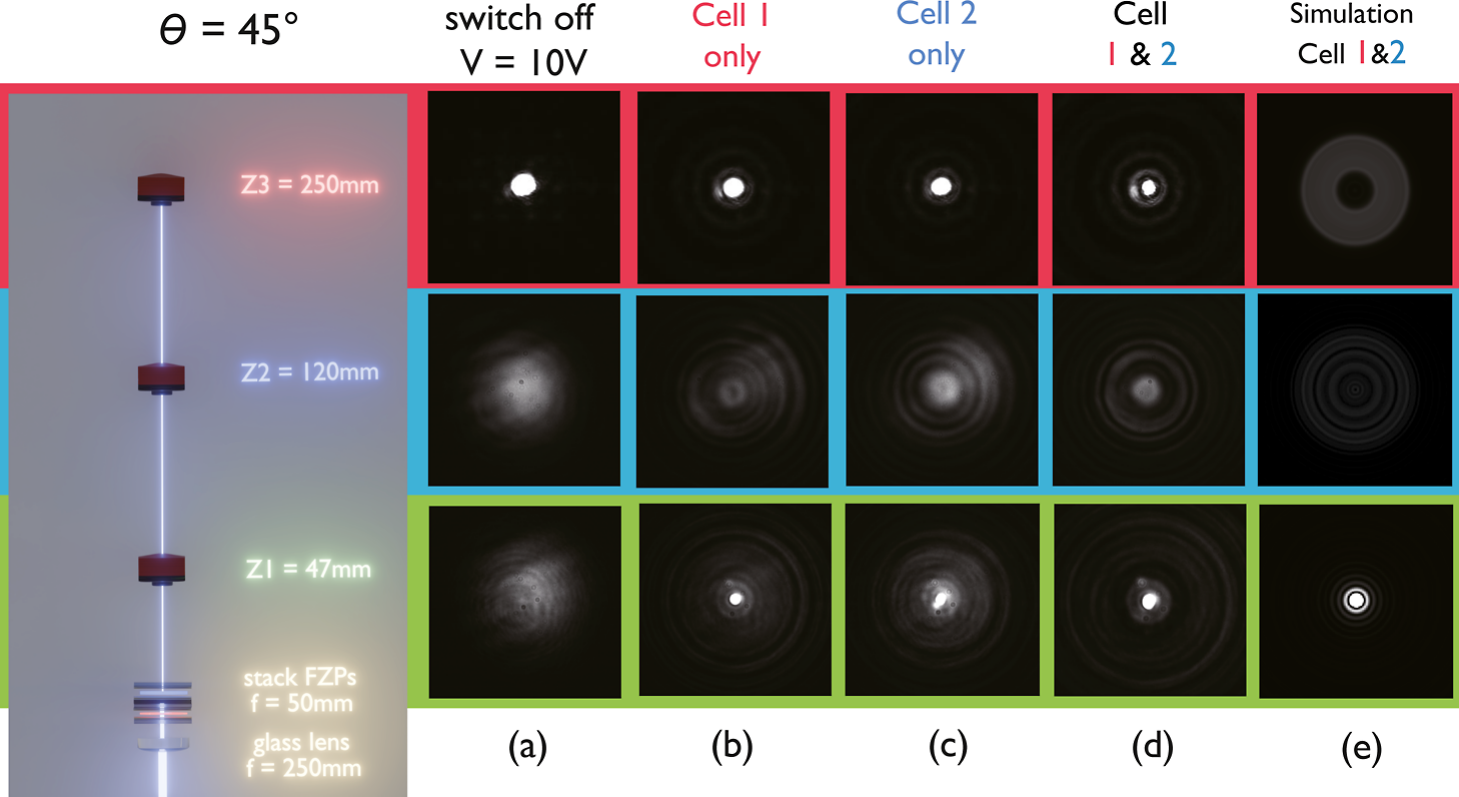
A varifocal optical system consisting of the stacked laser-written LC FZPs combined with a static glass lens in close proximity (shown on the left-hand side of the figure). The incident laser polarization was aligned at 45° to the rubbing direction of cell 1 for all measurements. Images recorded at the three different propagation distance *Z*: *Z*_1_ = 47 mm (green labels, bottom row), which is the designed focal length when the FZPs are *switched ON*, *Z*_2_ = 120 mm (blue label, middle row), which is not a designed focal length when the FZPs are either *switched ON* or *OFF*, and *Z*_3_ = 250 mm (red labels, top row), which is a designed focal length when the FZPs are *switched ON* for the following scenarios: (a) the stacked FZPs were both *switched OFF* with an applied voltage of 20 V; (b) cell 1 was switched on but cell 2 was *switched OFF*; (c) cell 2 was *switched ON,* but cell 1 was *switched OFF*; and (d) both cells were *switched ON*. (e) Simulations of the focusing effect at different focal planes when cells 1 and 2 were *switched ON*.

When the fixed lens and the LC FZPs are in close proximity (i.e., *d* is very small compared to the focal lengths), the equation can be approximated as

3 $$\frac{1}{f}\approx \frac{1}{f_{1}}+\frac{1}{f_{2}}$$

When the stacked FZPs were in the *OFF* state, they had an effective infinite focal length and thus did not modify the focusing of the integrated lens system. In the *ON* state, however, they enhance the focusing. Based on this concept, an additional refractive optical lens with *f* = 250 mm was installed in the characterization system for demonstrating varifocal operation. To clarify, combining one lens (*f* = 250 mm) and the stacked FZPs (*f* = 50 mm) provides two distinct focal planes located at either *f* = 47 mm, when the stacked FZPs are *switched ON* and *f* = 250 mm, when the stacked FZPs are *switched OFF*.

To demonstrate the varifocal ability, a CCD camera was placed at three different locations, shown in Figure 6, as follows: (1) where the combined focal plane was located *Z*_1_ = 47 mm, (2) somewhere in between the two focal planes, i.e. *Z*_2_ = 120 mm, and (3) at the focal length of the optical lens for when the FZPs were *switched OFF*, i.e. at *Z*_3_ = 250 mm. The linear polarization state of the incident laser beam was set to be aligned at 45° with respect to the rubbing direction of cell 1, ensuring that both cell 1 and cell 2 could focus the light.

By applying a high voltage to the stacked FZPs, it effectively results in the removal of one lens from the integrated lens system. In this case, the expected focal length would be *f* = 250 mm coming from the static glass lens, and consequently, the CCD camera did not capture any focusing effect at *Z*_1_ = 47 mm. In contrast, when the stacked FZPs were *switched ON* by applying 3.5 V, the focusing effect was clearly captured by the CCD camera at 47 mm, which demonstrated the idea of the integrated lens system. Conversely, when the CCD camera was placed at *Z*_2_ = 120 mm, which is away from the focus positions of the two “designed” focal lengths (*f* = 47 mm and *f* = 250 mm), the CCD camera did not capture any sharp focus as expected. Finally, when the CCD camera was placed at *Z*_3_ = 250 mm away from the integrated lenses, the sharp focusing was captured when the stacked FZP device was *switched OFF*, by applying high voltages to both cells, compared to no focusing in the same plane when the devices were *switched ON*.

It is important to note that even when the stacked FZPs were *switched ON*, resulting in an integrated lens focal length of *f* = 47 mm, there was still some leakage of light focused at a distance of *Z*_3_ = 250 mm, as depicted in Figure 6d. This residual focusing might be due to slight overfilling of the FZP active aperture by the incident Gaussian beam: the peripheral portion of the beam that lies outside the clear aperture is not phase-modulated by the FZPs and is, therefore, focused by the fixed refractive lens alone. In practice, this artifact could be mitigated by matching the beam diameter of the incident laser to the FZP clear aperture (e.g., using an iris or beam expander) so that the unmodulated periphery is suppressed. Moreover, the residual focusing might also be caused by the misalignment of the cell with the polarization of the incident laser; defocus arises from a mismatch between the actual beam waist and the design. These results demonstrate the switchable foci and the polarization independence of the FZPs, which is of potential interest in multiplane imaging for AR/VR devices.

### 2.3 On-Demand Holograms

Binary phase holograms are arbitrarily patterned phase devices designed to give a particular intensity profile in the far field. Using the technique described in the Methods section, computer-generated holograms (CGH) were designed to produce a double-headed arrow in the far-field (see Figure 7). The images were binarized for a high optical contrast. In addition to the LC cells being aligned such that their rubbing directions were orthogonal to one another, the holograms were also fabricated at 90° to one another, such that the hologram in cell 1 produced a horizontal arrow and the hologram in cell 2 generated a vertically aligned arrow. Characterization of the device used the same optical setup as that described in the Methods section.

**Figure 7:**
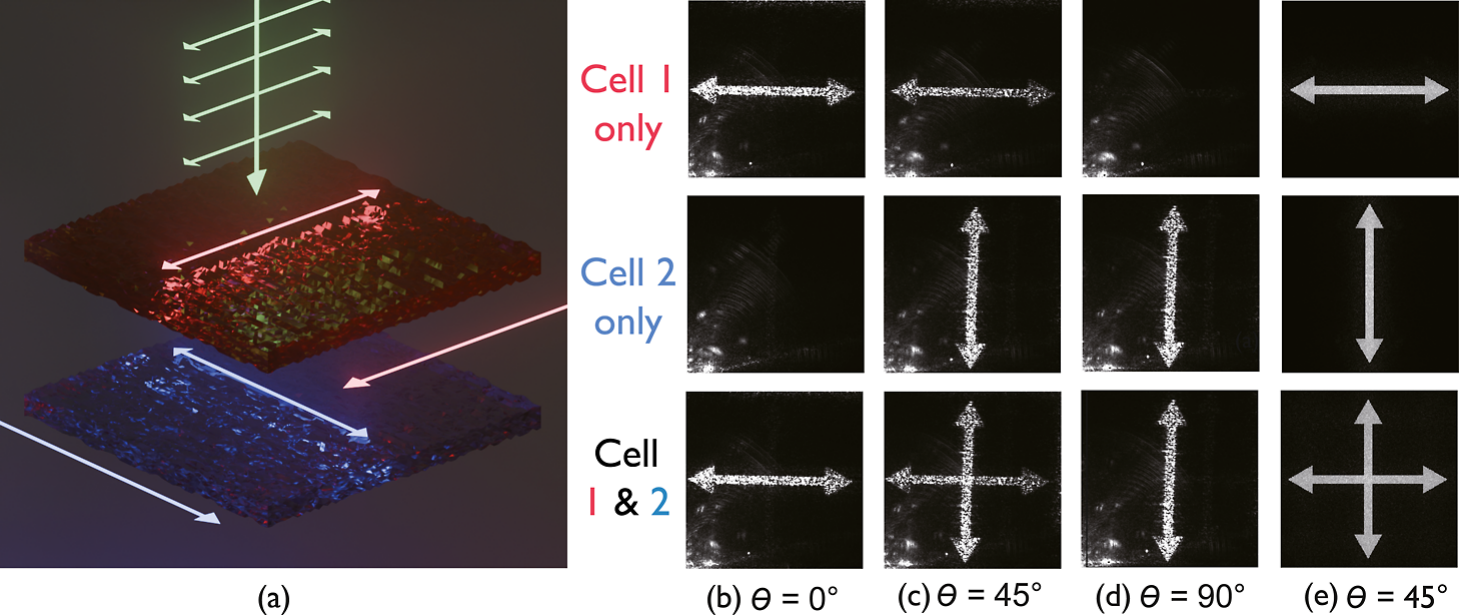
CGHs written into the LC cell 1 and cell 2 aligned orthogonal to one another. (a) Illustration of the configuration of both cells showing the corresponding alignments of the holograms and the rubbing directions. Far-field (replay) images for three different device configurations (top row—cell 1 *switched ON*, middle row—cell 2 *switched ON*, and bottom row—both cells activated) for the following orientations of the incident linear polarization state with respect to the rubbing direction of cell 1: (b) 0°, (c) 45°, and (d) 90°. (e) Simulation of the far-field images for an incident polarization aligned at θ = 45° relative to the rubbing direction of cell 1.

When only cell 1 was *switched ON* and the incident laser polarization was set to θ = 0° (Figure 7b), a bright horizontal double-headed arrow was observed in the far-field. This pattern grew dimmer when the polarization of the laser beam was rotated to 45°; however, the image remained consistent (Figure 7c). This is a result of cell 2 being *switched OFF*, such that only the light polarized along cell 1’s rubbing direction would be modulated. When the laser polarization was set to θ = 90° relative to the rubbing direction of cell 1, the horizontal double-headed arrow disappeared entirely.

When only cell 2 was activated, the vertical double-headed arrow was visible for incident laser polarization angles of θ = 45° and 90°. Finally, when both cells were simultaneously activated, the horizontal double-headed arrow was observed at 0° and 45°, while the vertical double-headed arrow appeared at 45° and 90°. Most importantly, whenever an unpolarized light source was incident on the device, both patterns would become visible simultaneously (Figure 7c).

The total diffracted power for the stacked laser-written CGHs was measured when both LC cells were *switched ON* with a total diffraction efficiency of 53.57% (9.73 μW), confirming that a significant portion of the incident optical energy is redistributed into the designed diffracted orders. Crosstalk is a common concern in multiplexed diffractive devices, particularly in polarization-selective holographic systems, where residual phase modulation from inactive channels can degrade reconstruction fidelity and order purity.

In this study, we experimentally evaluated the crosstalk behavior of the stacked polarization-multiplexed CGH. At an incident polarization of 45°, cell 1 (horizontal hologram channel) was *switched OFF,* while cell 2 (vertical hologram channel) was *switched ON* to examine whether any unintended horizontal hologram features were generated due to residual phase retardation in the inactive cell (see the Supporting Information, Figure S4). No visible horizontal hologram pattern was detected in the far-field under standard CCD acquisition settings (exposure time 0.4 ms), indicating negligible leakage from the vertical channel into the horizontal state. Even when the CCD exposure time was increased by more than 1 order of magnitude (up to 4.4 ms) to enhance sensitivity to weak light leakage signals, no discernible horizontal features were observed. These results demonstrate that the stacked LC hologram exhibits low crosstalk between orthogonal polarization channels, ensuring high order purity. Such polarization isolation is essential for multiplexed beam shaping, holographic data transmission, and display applications that require reliable and independent control of multiple information channels.

Furthermore, the order purity of the binary CGH was experimentally quantified by measuring the optical power contained in each diffraction order by using a calibrated photodiode. The first positive and negative orders were found to exhibit power purities of 9.22% and 7.94%, respectively (see Supporting Information Figure S5), when normalized to the total diffracted power across the −2 to +2 orders. These values are significantly lower than the theoretical efficiency of 40.5% expected for an ideal binary phase hologram. This discrepancy can be attributed to zero-order leakage, incomplete π phase modulation across the laser-written CGH, and fabrication-induced phase nonuniformity, which redistribute part of the optical power into the zeroth and higher unwanted orders. Nevertheless, the presence of well-defined first-order diffraction peaks confirms that the binary CGH operates as intended, albeit with room for improvement in the phase accuracy and modulation depth.

Operational stability is a crucial requirement for the practical deployment of LC optical elements in commercial photonic systems. To assess the temporal stability of the stacked LC device, both cells were *switched ON* and the optical power of the +first diffraction order from a binary phase grating was continuously monitored using a calibrated photodiode at 15 min intervals over a period of 3 days (see Supporting Information, Figure S6). The measured diffracted power exhibited only a small variation, decreasing from 1.49 to 1.45 μW, corresponding to a fluctuation of approximately 2.7% over the full measurement duration. Minor oscillations correlated with ambient temperature changes, indicating a degree of temperature sensitivity that is typical for nematic LC-based devices due to the thermo-optic dependence of the birefringence. Nevertheless, the overall variation of only 0.04 μW over 72 h demonstrates that the stacked device maintains stable optical performance under continuous operation, highlighting its suitability for long-term use in practical holographic and diffractive applications.

The above results underscore the potential to fabricate polarization-selective optical devices in which each polarization state could be addressed separately. While employing two orthogonally arranged holograms might intuitively suggest polarization-sensitive behavior, the use of unpolarized light would enable both patterns to be observed, demonstrating the feasibility of polarization-independent optical systems. In this experiment, we confirmed its polarization insensitivity using incident light with a polarization state of 45°, with respect to the rubbing directions of both cells.

It is important to note that LC gratings and polymer network gratings inherently exhibit limited diffraction efficiencies in the non-zeroth orders due to physical and fabrication-related constraints.^25–27^ In practice, scattering and absorption from the polymer microstructures, together with minor fabrication-induced phase errors and refractive index inhomogeneity, can further redistribute optical power away from the designed orders. This is common in LC-based diffractive optics and explains the nonideal efficiency observed in our measurements. Nonetheless, the stacked device still demonstrates an effective redistribution of optical power into the diffracted orders, confirming its practical phase modulation capability.

## 3 Conclusion

To conclude, we have demonstrated three different stacking LCOEs (diffraction gratings, binary FZPs and on-demand holograms). By demonstrating their optical behavior under various incident polarization states and different voltage conditions, we successfully demonstrated the ability for these stacked laser-written LC devices to modulate different polarization states of light to achieve the same far-field patterns or provide on-demand holographic patterns depending on the incident polarization state. Additionally, with the combination of a static refractive lens, it was shown that stacked FZPs can achieve switchable foci.

We have also quantitatively measured the power of these diffractive optic elements and their relative diffraction efficiency, proving that with the stacked configuration, an up to 80% increase of the optical efficiency can be realized for diffraction into the first order. For the laser-written CGH, the total diffraction efficiency was measured to be 53.57%. A stability measurement was also carried out over a period of 3 days, with continuous measurements for our stacked LC devices *switched ON*. The devices showed, overall, a small variation of 0.04 μW, which might be due to thermal fluctuation over this period of time. In the future, the assembly of a stacked device using a three-layer ITO sandwich structure would further increase the optical efficiency by preventing reflection and scattering losses when light penetrates the multiple layers. This will also reduce the total form factor further for potential commercially realizable optical elements.

## 4 Experimental Section

### 4.1 Device Fabrication

As shown in Figure 1, the stacked LC device was fabricated by stacking a pair of glass cells (Samsung ECB1X3-450) with rubbed polyimide alignment layers and a cell gap of 4.5 μm, maintained with glass spacer beads dispersed through the air gap. The glass substrates were coated with indium tin oxide (ITO). Indium solder was then used to attach wires to the ITO electrodes. Before assembly, the surfaces of the cells facing each other were cleaned with polyester swabs dipped in acetone to remove any dust or residue.

Cell 1 was stacked on top of cell 2, and was then aligned and stacked, ensuring that their optical axes were perpendicular. A drop of ultraviolet (UV)-curable glue (NOA61, viscosity 300 cps) was applied at the intersection of the two glass surfaces, allowing capillary action to draw the glue into the gap between the cells. Since the glue is viscous at room temperature and requires time to fill the gap uniformly, the assembly was placed in an oven at 50 °C to accelerate the process. The glue’s progress was carefully monitored to prevent it from seeping into the individual cells.

Once the gap was fully filled, the device assembly was placed in a UV box (CL-1000 UV cross-linkers, Analytikjena) at the maximum UV irradiation of 0.9999 kJ/cm^2^ and photopolymerized for 10 min to solidify the glue. These cells were then filled by capillary action with an LC mixture consisting of 79 wt.% E7 (Synthon Chemicals Ltd.), with 1.0 wt.% Irgacure 819 (Ciba-Geigy) photoinitiator and 20 wt.% RM257 (1,4-bis-[4-(3-acryloyloxypropyloxy) benzoyloxy]-2-methylbenzene (Synthon Chemicals Ltd.) reactive mesogen.

### 4.2 Two Photon Polymerization Direct Laser Writing

A TPP-DLW system was used to fabricate the desired patterns as needed at a voltage of 20 Vpp to ensure the director was aligned homeotropically during the laser writing process. The fabrication system used a Spectra-Physics Mai Tai Titanium-Sapphire laser (λ = 780 nm), delivering 100 fs pulses at an 80 MHz repetition rate. The laser beam was focused into the top cell in the stack using a 0.45 NA objective lens with 20× magnification. The sample was mounted on a three-dimensional translation stage constructed by integrating an Aerotech ANT95XY 2D translation stage with an ANT95v vertical translation stage. During the exposure, a square wave signal with a peak-to-peak voltage of 20 V and a frequency of 1 kHz was applied to the cell. This signal was generated by using a Tektronix AFG3021 signal generator and amplified by an FLC F10AD amplifier.

Following the fabrication of the first device, the stacked cells were flipped over so the second cell could be written into from above. This was done to minimize aberrations arising from the propagation through multiple glass layers. The structures were aligned between the two cells using the microscope built into the fabrication system by imaging the polymer structure in the bottom cell before being refocused into the top cell. As a 20 Vpp voltage was applied during the fabrication, this voltage must be applied post-fabrication to *switch OFF* the device. Similarly, a 3.5 Vpp voltage was applied to the device to *switch ON*.

For the diffraction gratings, the device was fabricated by using the TPP-DLW to polymerize a series of parallel *lines* when applying a 20 Vpp voltage with a line width of 1 μm and a line spacing of 4 μm, resulting in a total grating pitch of 5 μm. For the binary FZPs, λ = 632 nm was chosen to match the He–Ne laser system, with *f* = 10 cm, *n* = 10 to match the size of the laser beam. For the holograms, the pattern was calculated using the Gerchberg–Saxton Phase retrieval algorithm with a dimension of 500 × 500 μm^2^.

### 4.3 Far-Field Characterization

All devices were tested in the system shown in Figure 8. The illumination source in the system was a He–Ne laser emitting at 632.8 nm, which was the design wavelength for the devices shown here. The laser power was controlled with a neutral density filter and a linear polarizer. The output polarization was then set with a subsequent half-wave plate. A beam reducer was used to match the laser beam width to that of the device, and the device was positioned in the beam path with a 3-axis sample translation mount, and aligned with the laser using a microscope. The beam then passed through the device before being collected on a CCD camera in the far-field.

**Figure 8:**
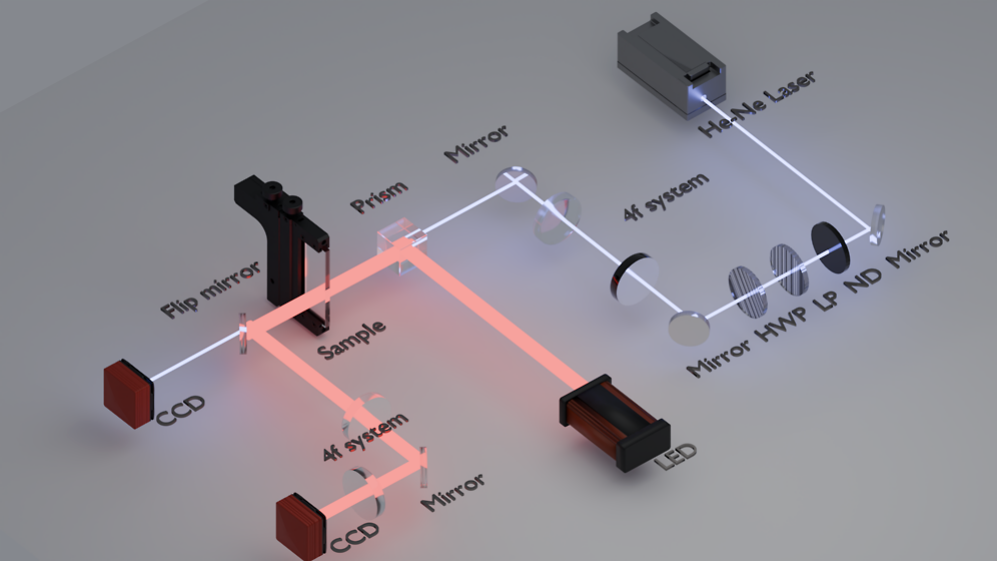
Schematic illustration of the optical system used for characterizing the polarization-independent laser-written LC optical elements.

For the varifocal lens experiment, the system was modified with a fixed glass lens in the same plane as the LC device, and the camera position was varied between the two design positions and one intermediate position. When the devices were *switched ON*, the diffractive elements fabricated in the cells could be used to diffract light because the remaining unpolymerized LC relaxed back to the planar orientation, while the polymerized LC remains vertically aligned. The difference in the orientation of the director in the polymerized and unpolymerized regions resulted in a retardance difference and therefore provided the phase pattern for light polarized parallel to the cell’s rubbing direction. The polarization angle was referenced in all cases to the rubbing direction of cell 1, which is the LC cell that the beam encountered first.

### 4.4 Diffraction Power Characterization

Quantitative diffraction power measurements were performed for both the binary diffraction gratings and the holograms fabricated using the stacked LC device architecture. A collimated He–Ne laser was beam-shaped to match the active aperture of the device using a spatial filter and a 4f optical system. The transmitted diffraction patterns were recorded in the far-field. The optical power was measured using a calibrated photodiode power sensor (Thorlabs S120VC, spectral range 200–1100 nm) connected to a Thorlabs Power Meter Interface to record the data.

To determine the total transmitted power *P*_total_, the photodiode was positioned immediately after the device, before significant free-space propagation occurred, such that all nondiffracted and diffracted components were collected simultaneously. Subsequently, to measure the nondiffracted (zeroth-order) component (*P*_0_), the photodiode was placed at the Fourier plane in the far-field and aligned to the optical axis. The total diffracted power *P*_diff_ was then calculated as

$$P_{diff}=P_{total}-P_{0}$$ 4

Using this method, the absolute and relative diffraction efficiencies were obtained for different diffraction orders. This procedure was identically applied to both the laser-written grating and hologram patterns to ensure a consistent comparison.

### 4.5 Stability Characterization

The stability measurements were undertaken in a similar way to that of the intensity recorded for the diffraction orders. The stacked laser-written devices were connected to a signal generator and subjected to an applied voltage throughout the measurement period. The photodiode was connected to a PC, and the measured power was recorded. Since all laser-written diffractive optic elements (diffraction gratings, FZPs, and holograms) were fabricated in the same stacked LC device, the first order from the diffraction gratings was captured using a photodiode throughout the measurement period to check if continuous illumination from the laser would degrade the polymer network inside the LC and thus reduce the optical efficiency of the stacked devices.

### 4.6 Simulations

All three of the device configurations demonstrated in this work (i.e., diffraction gratings, FZPs, and CGHs) have been simulated to verify the experimental results. Simulations were implemented in MATLAB, with computational domains discretized for high resolution. FFT algorithms were used for the efficient computation of light propagation, and the resulting intensity distributions were visualized to evaluate the optical properties of each system.

### 4.7 Diffraction Gratings

The diffraction gratings were modeled as arrays of parallel lines with periodic spacing, where the lines and background regions had refractive indices of *n*_lines_ = 1.515 and *n*_background_ = 1.730 at the designed wavelength of 633 nm, to simulate the ordinary refractive index of the polymerizable LC mixture and extraordinary refractive index, respectively. Phase modulation due to the refractive index contrast was computed using

5 $$\phi \left(x,y\right)=\frac{2\pi }{\lambda }\cdot \left(n\left(x,y\right)-n_{background}\right)\cdot d$$

where λ = 633 nm is the wavelength and *d* is the thickness of the grating medium. A Gaussian beam with a beam waist of *w*_0_ = 10 μm was applied to the grating, and the resulting field was propagated using the angular spectrum method

6 $$H\left(f_{x},f_{y}\right)=\exp\left(-ikz\sqrt{1-{\left(\lambda f_{x}\right)}^{2}-{\left(\lambda f_{y}\right)}^{2}}\right)$$

where $k=\frac{2\pi }{\lambda }$ . Intensity distributions were calculated at various distances to analyze the diffraction effects.

### 4.8 Binary FZPs

In this simulation, 632.8 nm was chosen to match the He–Ne laser system, with *f* = 10 cm, *N* = 10 to match the size of the laser beam. The focusing effect of FZPs was modeled by using a binary phase plate approach. A circular aperture was defined with alternating regions in phase of 0 and π to simulate constructive and destructive interference. The phase modulation was computed as

7 $$\phi \left(x,y\right)=k\left(\sqrt{f^{2}+x^{2}+y^{2}}-f\right)$$

where *f* is the focal length. A Gaussian beam was used as the input, and light propagation was simulated by using FFT-based Fresnel diffraction. Intensity profiles were visualized to evaluate the focusing performance, with postprocessing steps such as scaling and smoothing applied for clarity.

### 4.9 Holograms

To design phase-only holograms for TPP-DLW, we employed the Gerchberg–Saxton phase-retrieval algorithm.^28^ Each target intensity *I*_target_(*x*,*y*) was first down-sampled to a 500 × 500 grid, converted to an 8-bit grayscale, and binarized to enhance feature contrast. The iterative routine alternated between enforcing the image-plane amplitude constraint

8 $$E_{n}\left(x,y\right)\leftarrow \sqrt{I_{target}\left(x,y\right)}e^{i∠\left[E_{n}\left(x,y\right)\right]}$$

and propagating to the hologram plane via the angular-spectrum method

9 $$E_{n}\left(u,v\right)=F^{-1}\left\{F\left\{E_{n}\left(x,y\right)\right\}H\left(f_{x},f_{y}\right)\right\}$$

10 $$H\left(f_{x},f_{y}\right)=\exp\left(i2\pi z\sqrt{1/\lambda^{2}-f_{x}^{2}-f_{y}^{2}}\right)$$

followed by the phase-only projection

11 $$E_{n}^{\prime }\left(u,v\right)\leftarrow \exp\left(i∠\left[E_{n}\left(u,v\right)\right]\right)$$

and back-propagation. Simulations were performed at λ = 532 nm on a uniform grid with pixel pitch Δ*x* = 10 μm. After *N* = 100 iterations, convergence was quantified by the normalized root-mean-square error

12 $${NRMSE}_{n}=\frac{\sqrt{\sum_{x,y}{\left(|E_{n}{|}^{2}-I_{target}\right)}^{2}}}{\sqrt{\sum_{x,y}I_{target}^{2}}}$$

which typically fell below 5% by *n* ≈ 80. The final phase map ϕ_*N*_(*u*,*v*) was exported as an 8-bit PNG, with the pixel dimensions encoded in the filename to ensure full reproducibility.

## Supplementary Material



## Notes

Transmission as a function of voltage for the nematic LC mixture sandwiched in a glass cell, relative power of the diffraction gratings for the first five diffraction orders, variation in the normalized intensity for the first and third diffraction orders, far-field images of the laser-written holograms showing the amount of cross-talk, purity of the diffraction orders, and power recorded in the first diffraction order over a period of 3 days (PDF)
