# Supplementary material for: Polarization-Independent and Electrically Tunable Polymerized Liquid Crystal Optical Elements
Source: ACS Photonics. 2025 Dec 9;13(1):47–57. doi: 10.1021/acsphotonics.5c01416 (PMC12784407; doi:10.1021/acsphotonics.5c01416)
Supplement: Supplementary file 1 [file ph5c01416_si_001.pdf]

Supporting Information

Polarization Independent and Electrically-tunable Polymerized Liquid Crystal Optical Elements

Zhiyu Xu, Camron Nourshargh, Waqas Kamal, Alec Xu, Steve J Elston, Martin J. Booth, and Stephen M. Morris∗

Department of Engineering Science, University of Oxford, Oxford, OX1 3PJ, UK

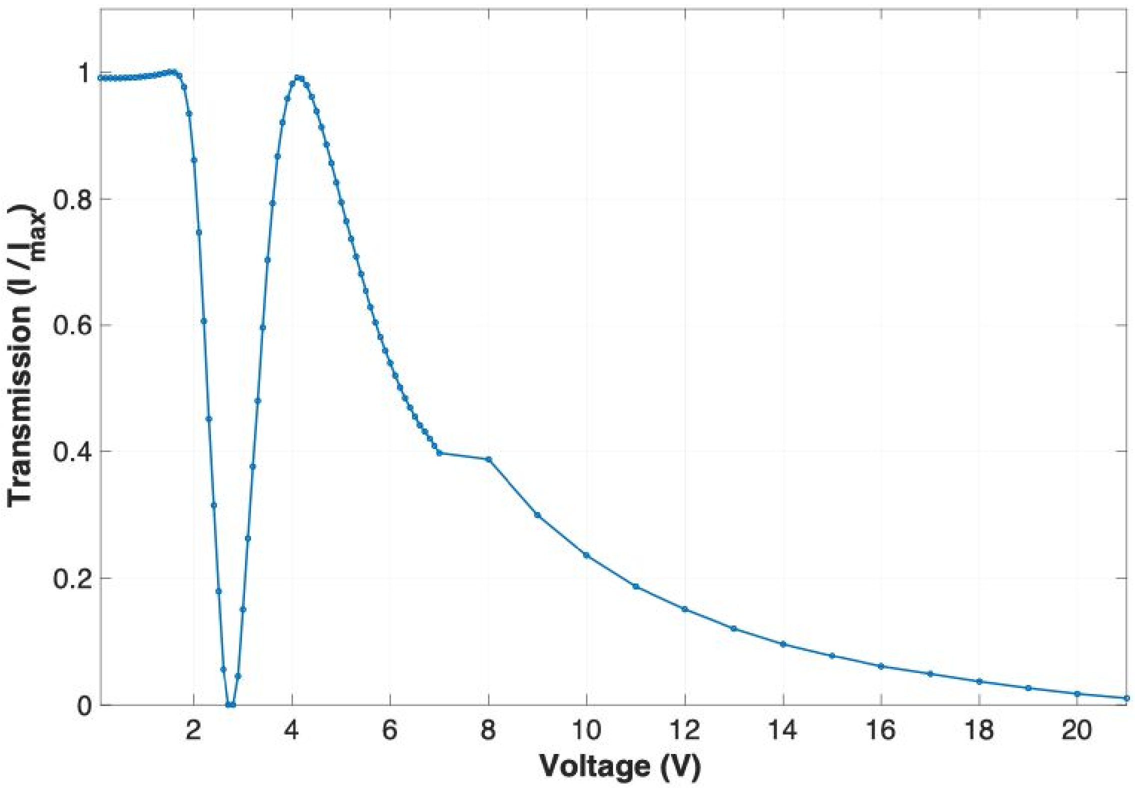

Figure S1. Transmission as a function of the applied voltage (Vpp) for a 5 micron thick LC layer of the nematic mixture E7 (Synthon Chemicals Ltd.) sandwiched in a glass cell. The transmission was recorded for a bipolar square wave electric field at a frequency of 1 kHz. The solid line is to guide the eye.

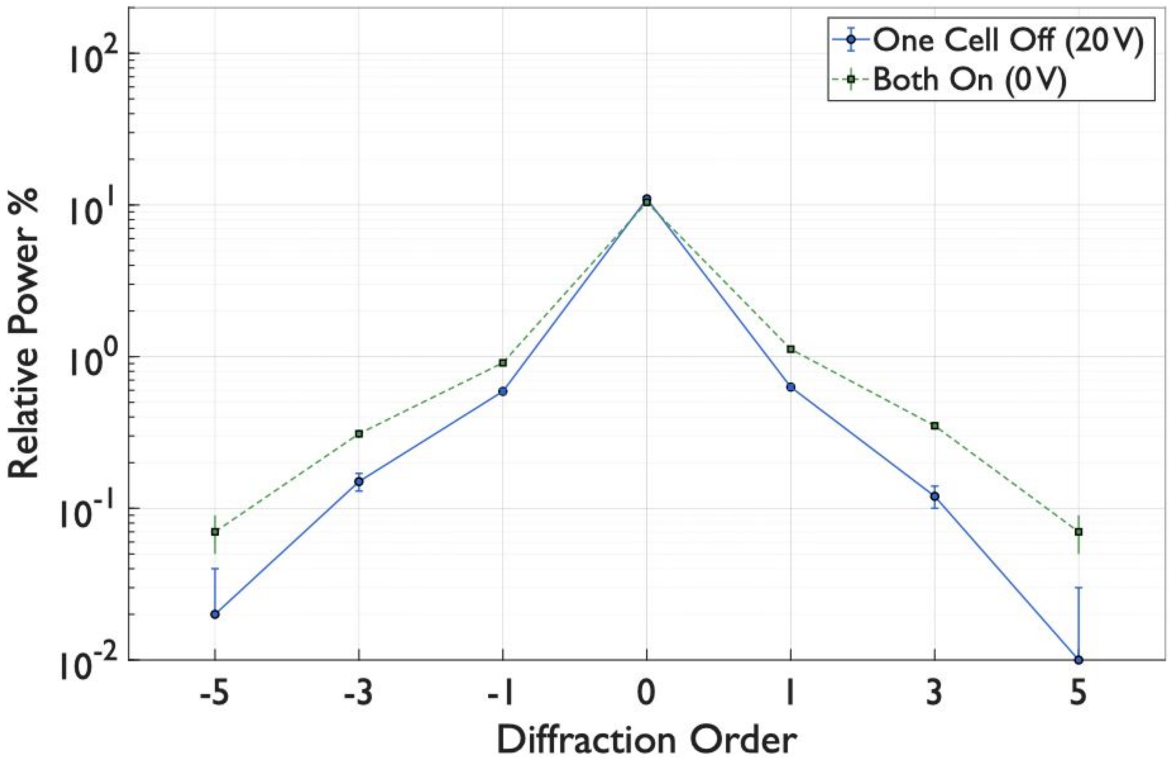

Figure S2. Relative power recorded for the first ±5 diffraction orders from the stacked TTP-DLW fabricated LC gratings. Results are presented for the case when only one LC cell is switched ON (blue circle data points) and when both LC cells are switched ON (green square data points). The solid and dashed lines are included to guide the eye.

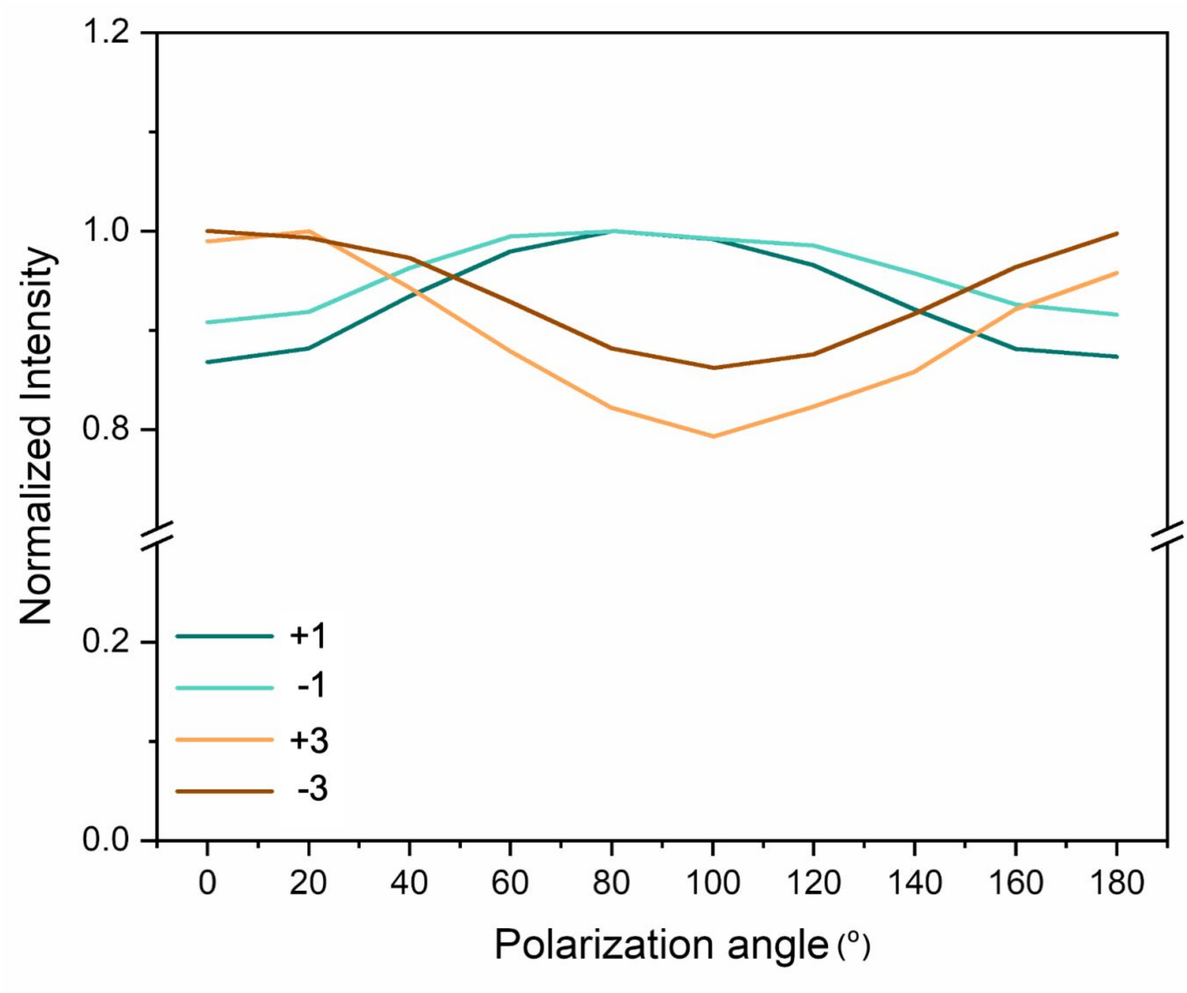

Figure S3. Variation in the normalized intensity for the ±1 and ±3 diffraction orders as a function of the angle between the incident linear polarization and the rubbing direction of the alignment layers. A halfwave plate was used to rotate the plane of polarization of the incident laser.

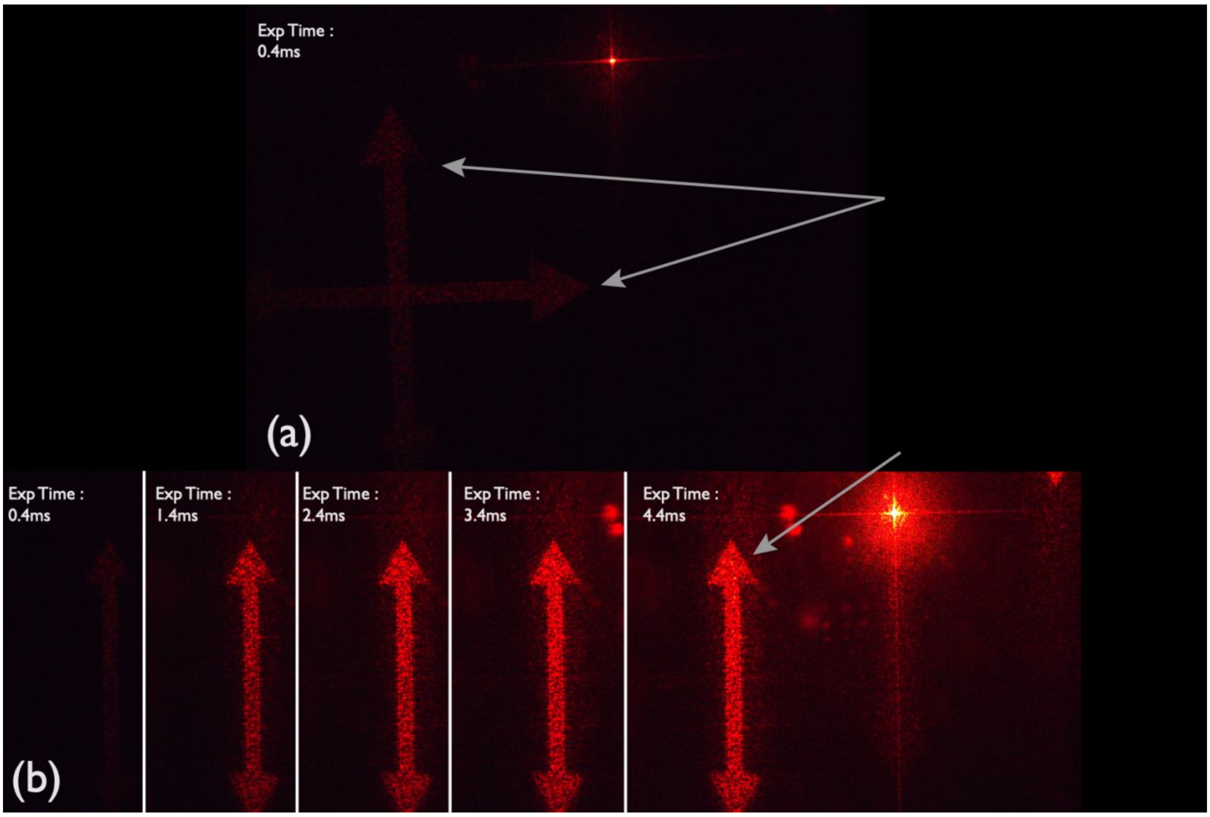

Figure S4. Far-field images for the laser-written computer-generated holograms showing the amount of crosstalk for incident linear polarized light oriented at 45° with respect to the alignment layer of Cell 1. (a) Far-field images when both Cell 1 and 2 are switched ON and the orthogonal arrow pattern appeared. (b) Far-field images when only Cell 1 was switched ON. Multiple images are shown for increasing exposure time (shown in the top left of each image) in order to exam the effect of crosstalk.

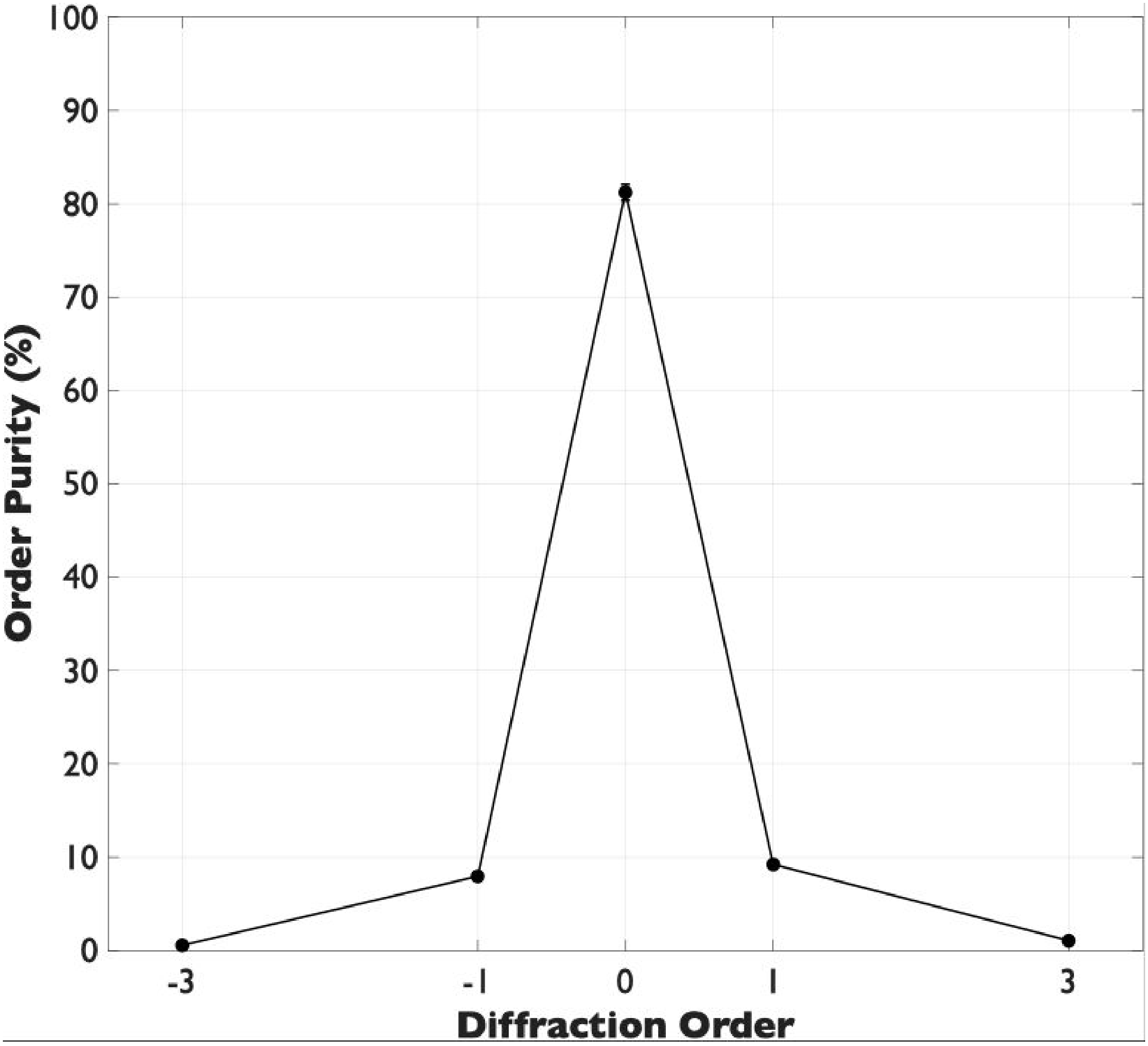

Figure S5. Purity of the diffraction orders in the stacked TPP-DLW fabricated LC device. The order purity is defined as the ratio of the intensity of a specific diffraction order to the total measured intensity across all considered orders within the region of interest. The solid line is to guide the eye.

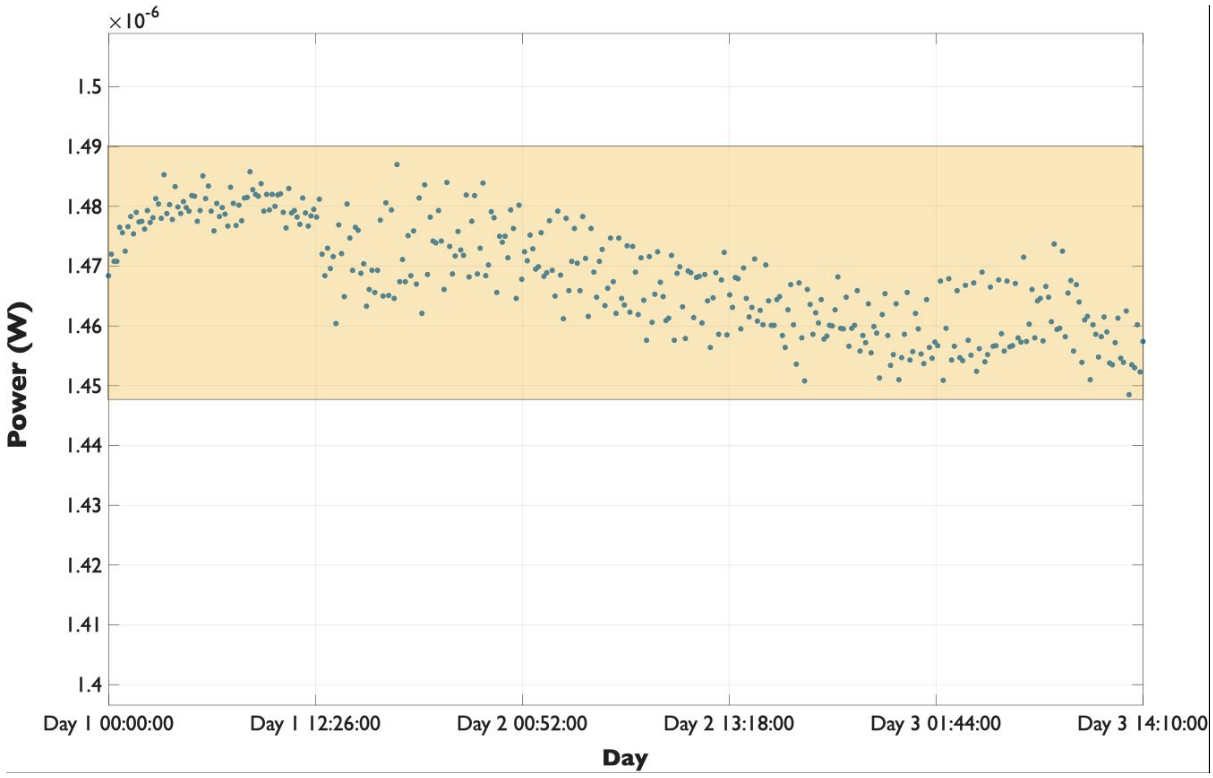

Figure S6. Power recorded in the 1st diffraction order in the laser-written diffraction grating as a function of time. A photodiode was used to record the power.
